# Endothelial Cell Inflammation and Barriers Are Regulated by the Rab26-Mediated Balance between *β*2-AR and TLR4 in Pulmonary Microvessel Endothelial Cells

**DOI:** 10.1155/2019/7538071

**Published:** 2019-04-28

**Authors:** Huaping Chen, Ming Yuan, Chunji Huang, Zhi Xu, Mingchun Li, Chun Zhang, Zhan Gao, Mingzhou Zhang, Jiancheng Xu, Hang Qian, Jiegen You, Binfeng He, Guansong Wang, Mingdong Hu

**Affiliations:** ^1^Institute of Respiratory Diseases, Xinqiao Hospital, Third Military Medical University, Chongqing 400037, China; ^2^Basic Medical College, Third Military Medical University, Chongqing 400038, China; ^3^Jiangxi Academy of Medical Sciences, Nanchang City, Jiangxi Province 330006, China

## Abstract

Rab26 GTPase modulates the trafficking of cell surface receptors, such as G protein-coupled receptors including *α*2-adrenergic receptors in some cell types. However, the effect of Rab26 on *β*2-adrenergic receptor (*β*2-AR) trafficking or/and Toll-like receptor 4 (TLR4) expression in human pulmonary microvascular endothelial cells (HPMECs) is still unclear. Here, we investigated the role of Rab26 in regulating the expression of *β*2-ARs and TLR4 in HPMECs and the effect of these receptors' imbalance on endothelial cell barrier function. The results showed that there was unbalance expression in these receptors, where *β*2-AR expression was remarkably reduced, and TLR4 was increased on the cell membrane after lipopolysaccharide (LPS) treatment. Furthermore, we found that Rab26 overexpression not only upregulated *β*2-ARs but also downregulated TLR4 expression on the cell membrane. Subsequently, the TLR4-related inflammatory response was greatly attenuated, and the hyperpermeability of HPMECs also was partially relived. Taken together, these data suggest that basal Rab26 maintains the balance between *β*2-ARs and TLR4 on the cell surface, and it might be a potential therapeutic target for diseases involving endothelial barrier dysfunction.

## 1. Introduction

Acute lung injury (ALI) carries high mortality rates worldwide due to the overwhelming pulmonary inflammation and loss of endothelial barrier function induced by cell hyperpermeability [1, 2]. Human pulmonary microvascular endothelial cells (HPMECs) are an important component of the vascular endothelial barrier, which is a semiselective permeable barrier that regulates the exchange of blood, fluid, proteins, and electrolytes across the vascular wall [3]; furthermore, Rab proteins play an important role in mediating endothelial barrier function and inflammation in acute respiratory distress syndrome (ARDS) [4–6].

Rab26 is a new member of the Rab GTPase family which controls cellular survival, homeostasis, and the stress response [7]. In an early study, Rab26 was found on secretory granules from parotid acinar cells [8]. Previous studies also demonstrated that Rab26 plays an important role in regulating *α*2-AR transport from the Golgi to the membrane [9], directing lysosome trafficking [10] and inducing autophagy [11]. Furthermore, as shown in our previous study, the GTPases Rab1 and Rab5a control permeability by regulating the localization and trafficking of *β*-AR in HPMECs [4, 5]. *β*-AR is a prototypic G protein-coupled receptor (GPCR) and is involved in maintaining vascular function and barrier integrity [12]. Another study has shown that norepinephrine-induced IL-6 release, which contributes to inflammatory responses, is primarily controlled through *β*2-AR in human dermal PMECs [13]. Our previous studies suggested that *β*2-AR may play a role in LPS-induced cell hyperpermeability [4]. Nevertheless, whether Rab26 modulates *β*2-AR trafficking to regulate the permeability and inflammatory response of HPMECs remains unclear.

Although GPCRs (*β*2-AR) protect the endothelial barrier and cell structure and function against damage from LPS-induced hyperpermeability at the HPMEC surface [12], Toll-like receptors (TLRs), especially TLR4 which is an essential contributor to inflammation in ALI/ARDS, are the main effectors of the LPS-induced inflammatory response. TLR4, which also localizes to the cytoplasmic membrane, recognizes pathogen-associated molecular patterns and reacts to damage-associated molecular patterns to promote the secretion of massive amounts of endogenous antiseptic inflammatory molecules in response to injuries at the cell surface [14]. The data show that TLR4 activation promotes lung inflammation, thereby aggravating lung injury; in contrast, TLR4 inhibition prevents lung edema and actin cytoskeleton rearrangement in HPMECs [15]. Interestingly, Rab26 modulates the permeability and apoptosis of HPMECs by manipulating the pathways downstream of TLR4 signaling [16]. Therefore, the regulation of both *β*2-AR and TLR4 involves Rab26; however, the mechanism by which Rab26 controls these two receptors and the molecular relationship between these proteins in HPMECs remain poorly understood.

In this study, we investigated whether Rab26 is involved in LPS-induced endothelial barrier dysfunction. By overexpressing Rab26, we determined its ability to mediate endothelial permeability. We also explored the relationship between *β*2-AR and TLR4 in LPS-induced hyperpermeability and inflammatory responses and examined the role of Rab26 in regulating receptor balance in HPMECs. Our results demonstrated that Rab26 is a key mediator of LPS-induced vascular hyperpermeability and plays a novel role in regulating the imbalance of *β*2-AR and TLR4 in HPMECs.

## 2. Materials and Methods

### 2.1. Reagents and Antibodies

The following plasmids were gifted from Guangyu Wu (Department of Pharmacology and Toxicology, Medical College of Georgia, Augusta University, GA, USA): Rab26 wild type (Rab26^WT^) and its mutants Rab26 Q123L (Rab26^QL^, GTP-bound form) and Rab26 N177I (Rab26^NI^, guanine nucleotide binding-deficient); they were cloned into vector pDsRed-monomer-C1 and HA-pcDNA3.1- [9] (Fig.  S1). The sequences of *β*2-AR siRNA (5′-CUCAAGACGUUAGGCAUCATTUGAUGCCUAACGUCUUGAGTT-3′) and TLR4 siRNA (5′-GGGCUUAGAACAACUAGAATTUUCUAGUUGUUCUAAGCCCTT-3′) were synthesized by Sangon Biological Engineering (Shanghai, China). The primary HPMECs were obtained from CELLBIO (Shanghai, China). Endothelial cell medium (ECM), Opti-MEM, and endothelial cell growth supplement (ECGS) were obtained from ScienCell (CA, USA). The ViaFect™ transfection reagent was obtained from Promega (WI, USA), and the X-tremeGENE siRNA transfection reagent was purchased from Roche (Basel, Switzerland). Isoproterenol (ISO), lipopolysaccharide (LPS, from E. coli 0111:B4), Tween 20, 4% paraformaldehyde (PFA), and 0.1% Triton X-100 were obtained from Sigma (MO, USA). The M-PER Mammalian Protein Extraction Reagent and Membrane Protein Extraction kit were purchased from Thermo Fisher Scientific (MA, USA). Fetal bovine serum (FBS) and 0.25% trypsin were obtained from Life Technologies (CA, USA). Polyvinylidene difluoride (PVDF) membranes and the enhanced chemiluminescence (ECL) reagent were purchased from Millipore (MA, USA). 4′,6-Diamidino-2-phenylindole (DAPI) was obtained from Santa Cruz Biotechnology (CA, USA). Transwell inserts were purchased from Costar (MA, USA), and biotin-conjugated bovine serum albumin was purchased from Thermo Fisher Scientific (MA, USA). All of the reagents were commercially available and used as received. The primary antibodies used in this study were Rab26 [16], *β*2-AR [17], GAPDH, and Na^+^-K^+^-ATPase [18] obtained from Abcam (all rabbit monoclonal, Cambridge, MA, USA); TLR4 [16] obtained from Boster (rabbit monoclonal, Beijing, China); human *β*2-AR obtained from Abcam [19]; and human TLR4-PE [16] obtained from eBioscience (MA, USA) and for use in flow cytometry. The monoclonal secondary antibodies used include the following: Alexa Fluor 594 (goat anti-rabbit IgG) [20] and FITC-labeled anti-rabbit IgG antibody (Abcam, MA, USA). We also used horseradish peroxidase- (HRP-) coupled [16] goat anti-rabbit purchased from Zhongshan Jinqiao Biological Technology (Beijing, China).

### 2.2. Cell Culture

HPMECs were cultured in ECM with 2% ECGS and 10% FBS in a 95% humidified atmosphere with 5% CO_2_ at 37°C. When the cells reached 90% confluency, they were trypsinized.

### 2.3. Confocal Microscopy and Image Analysis

HA-Tagged-Rab26 plasmids were prepared. The experiments were divided into five groups, namely, the control (Opti-MEM), mock (empty vector)/LPS, Rab26^WT^/LPS, Rab26^QL^/LPS, and Rab26^NI^/LPS. HPMECs were grown on cover slips in a 24-well plate with 0.5 × 10^5^ cells each well for 24 h and transfected with the ViaFect™ transfection reagent (4 *μ*L) and plasmid (1 *μ*g) for 6 h, respectively. After 72 h, the cells were treated with 1 *μ*g/mL LPS for 6 h. The cells were washed with PBS, permeabilized with 0.1% Triton X-100 for 10 min, fixed in 4% paraformaldehyde at 4°C for 30 min, and blocked with PBS containing 2% BSA for 1 h at room temperature. Subsequently, the cells were incubated with anti-Rab26 (1 : 200 dilutions) at 4°C overnight. The cells were incubated with secondary antibodies including Alexa Fluor 594 at 1 : 300 dilutions for 1 h at room temperature. These immunofluorescence images were captured by a confocal laser scanning microscope (Leica TCS SP5, Heidelberg, Germany).

### 2.4. Flow Cytometry Analysis for the Expression of Receptors on the Cell Membrane

The materials and treatment are as described in Section 2.3. After 72 h transfection, the cells were treated with LPS for 6 h. The cells were harvested with 0.25% trypsin, centrifuged for 10 min at 1000 rpm, and washed with equithermic PBS. Then, they were incubated with anti-human-*β*2-AR or anti-human TLR4-PE for 30 min at 4°C. To measure the *β*2-AR levels, the cells were washed with FACS buffer again and labeled with FITC-labeled secondary antibody at 4°C for 10 min. Subsequently, the cells were washed with PBS and centrifuged, and the cell surface expression of receptors was measured via the FITC or PE fluorescence intensity on a flow cytometer (Becton Dickinson, CA, USA).

### 2.5. Treatment with ISO or LPS

The experiments were divided into four groups, namely, blank, ISO, LPS, and ISO/LPS. The protocol was performed as previously described [5]. The cells were cultured in 6-well plates and treated with LPS (1 *μ*g/mL) or ISO (1 *μ*M) or pretreated with LPS for 6 h followed by ISO for 1 h at 37°C. Finally, the cells were used in subsequent experiments.

### 2.6. Transfection of siRNA

The experiments were divided into four groups, namely, negative control (NC), *β*2-AR siRNA, TLR4 siRNA, and *β*2-AR siRNA/TLR4 siRNA. The cells were seeded in 6-well plates at a density of 2 × 10^5^ cells/well and precultured for 24 h. The siRNA (100 nM) and siRNA transfection reagent (2 *μ*L) were diluted in 200 *μ*L of Opti-MEM in separate tubes, following the protocol from a previous study [6]. The tubes were combined for 5 min, mixed, and incubated for 20 min at room temperature. Then, the mixture was added into the culture medium. After 48 h, the cells were collected for subsequent experiments.

### 2.7. Western Blot Analysis of Rab26, TLR4, and *β*2-AR Protein Expression

Western blots were performed as previously described [10]. Cells were treated according to the requirements of each group, and then, they were collected in cold PBS and centrifuged at 1500 rpm for 10 min at 4°C. We extracted the total protein and membrane protein fractions. The procedure of membrane protein extraction was the following: Cultured cells were collected and resuspended with cell wash solution. They were then centrifuged at 1500 rpm for 10 min at 4°C. After removing the supernatant, HPMECs were suspended and incubated with 0.75 mL permeabilization buffer for 10 min at 4°C, then centrifuged at 16000 rpm for 15 min at 4°C. The supernatant was collected. Next, the supernatant was incubated with 0.5 mL solubilization buffer for 30 min at 4°C and centrifuged again. Finally, the supernatant was collected and measured with a protein analyzer (Thermo Scientific, USA). Each sample (50 *μ*g) was separated by sodium dodecyl sulfate-polyacrylamide gel electrophoresis (SDS-PAGE), and the proteins in the gel were transferred onto PVDF membranes for 1 h. Then, the membranes were blocked with 5% nonfat milk for 60 min and were incubated overnight with antibodies against Rab26 (1 : 500 dilutions), *β*2-AR (1 : 500 dilutions), TLR4 (1 : 200 dilutions), GAPDH (1 : 1000 dilutions), or Na^+^-K^+^-ATPase (1 : 500 dilutions). The blots were washed with TBST (3 × 5 min) and incubated with a secondary HRP-conjugated antibody (goat anti-rabbit) at 1 : 5000 dilutions with 5% nonfat milk for 1 h at room temperature. Finally, the protein signals were detected with an ECL detection system, and the results were analyzed with the Quantity One software [4]. GAPDH or Na^+^-K^+^-ATPase was used as the standard to verify equivalent loading, and the results were expressed as the percentage of GAPDH or Na^+^-K^+^-ATPase.

### 2.8. Endothelial Cell Permeability Assay

The permeability of HPMEC monolayers was determined by diffusion of biotin-BSA, following previous description [5]. Briefly, the HMPECs were seeded on a 12-well plate (1 × 10^3^ cells each well) lined with polycarbonate filters (0.4 *μ*m) for 24 h. After treatment, biotin-BSA (500 *μ*g/mL) was added into the upper chamber wells for 30 min; then, 100 *μ*L lower chamber medium was collected to detect the biotin-BSA concentration. Finally, the concentration of biotin-BSA in the media was measured by enzyme-linked immunosorbent assays (ELISA); the protocol was performed as previous study [4, 21].

### 2.9. Cytokine Secretion Assay

Cell supernatants were collected and used to measure TNF-*α*, IL-6, IL-8, and IL-17A with ELISA kits (Thermo Scientific, Rockford, IL). The experiments were conducted according to the instructions of the ELISA kits.

### 2.10. Statistical Analysis

The data are presented as the mean ± SD, and each experiment was repeated at least three times. Two-tailed Student's *t*-test was used for experiments consisting of two groups, and one-way analysis of variance (ANOVA) was used for experiments consisting of more than two groups. The results were deemed statistically significant when *p* < 0.05.

## 3. Results

### 3.1. Rab26 Regulates LPS-Induced Hyperpermeability and Inflammation in HPMECs

**Figure 1:**
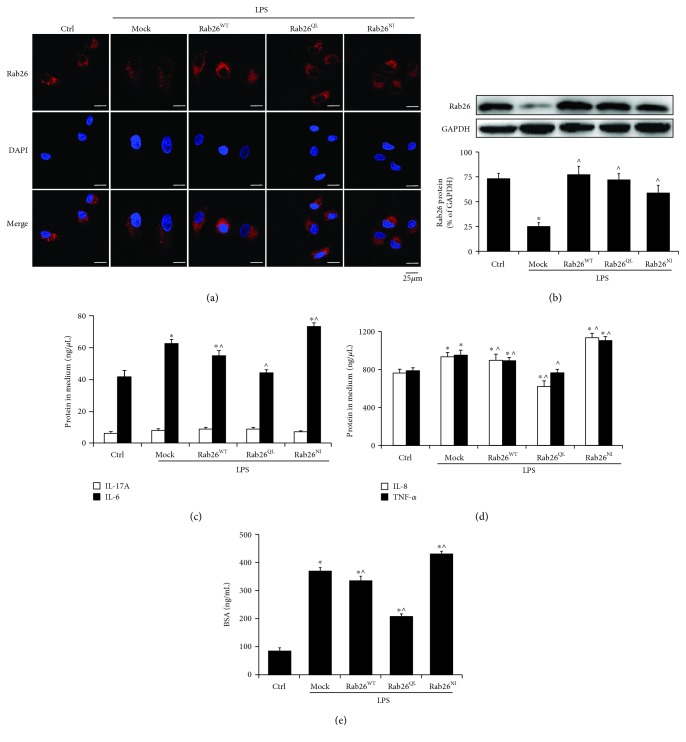
Rab26 regulates LPS-induced inflammation and hyperpermeability in HPMECs. Mock: vector; Rab26^WT^: Rab26 wild type; Rab26^QL^: Rab26 Q123L; Rab26^NI^: Rab26 N177I. (a) Confocal microscopy analyses of the Rab26 protein expression profiles. HPMECs were transfected with HA-tagged Rab26 plasmids for 72 h and treated with LPS (1 *μ*g/mL) for 6 h. Then, the cells were incubated with the Rab26 (1 : 200 dilution) antibody followed by labeling with Alexa Fluor 594-conjugated secondary antibody (1 : 300 dilution). Red: Rab26; blue: cell nuclei. Scale bars: 25 *μ*m. (b) Western blots showed the Rab26 expression after transfection with the HA-tagged Rab26 plasmids and LPS treatment. The histograms show the quantitative analysis of the protein expression levels which were normalized to GAPDH expression. (c, d) Expression of inflammatory factors after transfection with the Rab26 plasmids by ELISA (IL-6, IL-17A, IL-8, and TNF-*α*). (e) BSA permeability analysis of the HPMECs transfected with the DsRed-tagged Rab26 plasmids and treated with LPS. The data are the means ± SD (*n* = 3). ^∗^ *p* < 0.05 versus the control group; ^^^ *p* < 0.05 versus the mock group.

Our previous study found that overexpression of Rab26 partially inactivates LPS-induced TLR4 signaling and attenuates the hyperpermeability of HPMECs [16]. In this study, we determined the effect of transient Rab26 expression on permeability and LPS-induced inflammation. The analysis of DsRed-tagged Rab26 transfection efficiency is shown in Fig.  S2. As shown in Figure 1(a), Rab26 (red) was mainly localized in the perinuclear region, and LPS exposure reduced the Rab26 protein levels. In contrast, Rab26 expression was significantly increased after transfection with Rab26 plasmids compared with the mock group. Thus, the data revealed that the LPS-induced downregulation of Rab26 was attenuated by Rab26 plasmids (*p* < 0.05) (Figure 1(b), Fig.  S3).

Then, we observed the effect of Rab26 on inflammation and permeability. The results indicated that upregulation of Rab26 decreased the expression of the inflammatory mediators IL-6, IL-8, and TNF-*α* (*p* < 0.05), but it did not significantly change IL-17A expression among these groups (*p* > 0.05) (Figures 1(c) and 1(d)); the possible explanation was that IL-17A was primarily secreted by T helper 17 cells, which were lightly produced in endothelial cells. Next, we found that the cell permeability was obviously increased after LPS stimulation, compared with the control. But Rab26^QL^ attenuated LPS-induced hyperpermeability, compared with the mock. In contrary, Rab26^NI^ significantly exacerbated the endothelial hyperpermeability, compared with the mock (Figure 1(e)). The result was in agreement with our previous study that Rab26^QL^ (GTP-bound form) improved adherens junction integrity and store cellular permeability by activating autophagy, and Rab26^NI^ promoted endothelial barrier damage [22]. These data demonstrated that Rab26 plays a crucial role in strengthening endothelial barrier function.

### 3.2. Rab26 Regulates the Imbalance of *β*2-AR and TLR4 from LPS-Induced Injury in HPMECs

**Figure 2:**
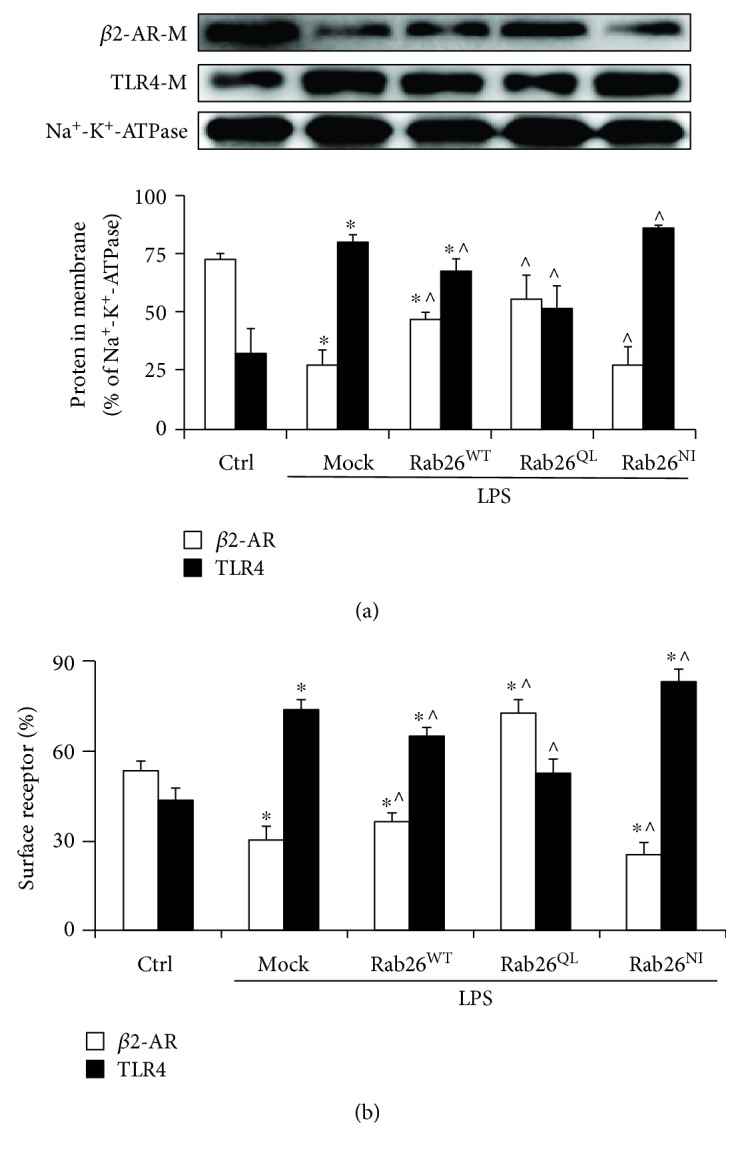
Rab26 regulates LPS-induced imbalance of *β*2-AR and TLR4. (a) Effect of Rab26 on the cell surface expression of proteins in HPMECs. HPMECs were transfected with DsRed-tagged Rab26 plasmids for 72 h and treated with LPS (1 *μ*g/mL) for 6 h; then, Western blot analyzed the membrane protein expression. The histograms show representative *β*2-AR, TLR4, and Na+-K+-ATPase expression and quantitative data normalized to Na+-K+-ATPase, respectively. (b) HPMECs were pretransfected with HA-tagged Rab26 plasmids for 72 h and treated with LPS (1 *μ*g/mL) for 6 h. Flow cytometry detected the expression of cell surface receptors. The data are the means ± SD (*n* = 3). ^∗^ *p* < 0.05 versus the control group; ^^^ *p* < 0.05 versus the mock group.

Based on the previous results, we confirmed that Rab26 regulates the LPS-induced inflammation and permeability of HPMECs; so, we next sought to determine the potential mechanism. To characterize the functional role of Rab26 in regulating *β*2-AR and TLR4 expression at the cell surface, we sought to determine the effect of Rab26 on the membrane expression of *β*2-AR and TLR4. The result clearly indicated that the expression of *β*2-AR was obviously decreased, but that of TLR4 was markedly increased after treatment with LPS in mock, compared with the control; however, Rab26^QL^ significantly promoted the expression of *β*2-AR on the plasma membrane and inhibited the TLR4 expression on the cell surface, compared to mock; on the contrary, Rab26^NI^ reduced the *β*2-AR expression and enhanced TLR4 expression, compared to mock (*p* < 0.05) (Figure 2(a), Fig.  S4). Consistent with this finding, receptor expression followed this trend (Figure 2(b)). These data preliminarily indicated that the Rab26 level is required for enhancing *β*2-AR and inhibiting TLR4 expression on the cell surface, and there is a potential imbalance between *β*2-AR and TLR4 that is controlled by Rab26.

### 3.3. Effect of Agonists on *β*2-AR and TLR4 in HPMECs

**Figure 3:**
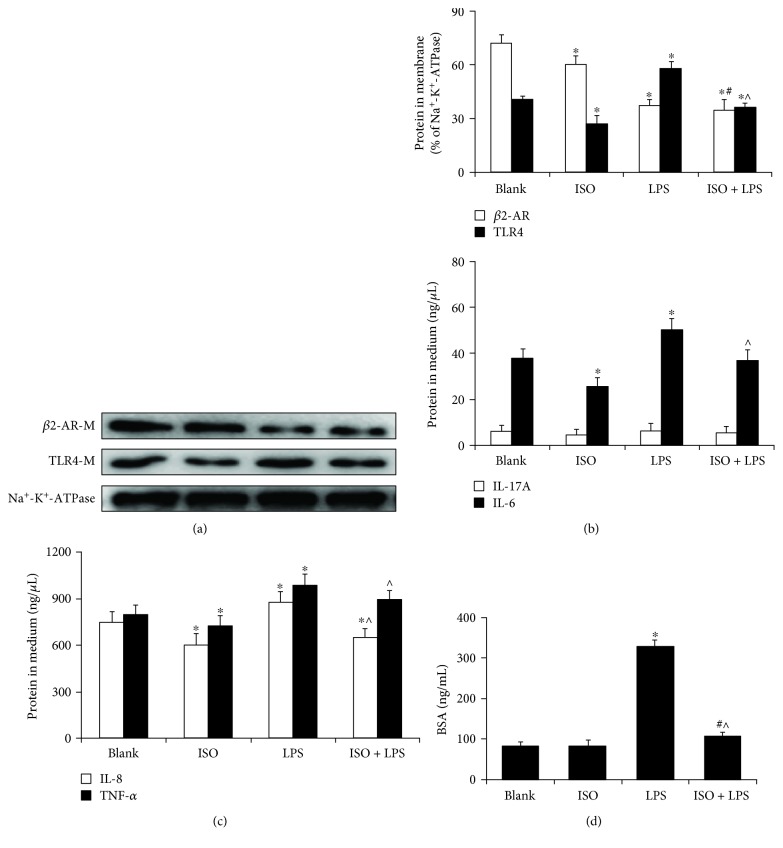
Effect of agonists on the expression of *β*2-AR and TLR4 in HPMECs. (a) Effect of agonists on the cell surface expression of proteins in HPMECs. The representative blots show the expression of *β*2-AR (A, upper), TLR4 (A, middle), and Na^+^-K^+^-ATPase (A, lower). The histograms illustrate the quantitative analysis of the *β*2-AR and TLR4 protein levels normalized to Na^+^-K^+^-ATPase. (b, c) Expression of inflammatory factors (IL-6, IL-17A, IL-8, and TNF-*α*) after treatment with ISO and LPS. (d) Effect of ISO and LPS treatment on monolayer permeability. The data are the means ± SD (*n* = 3). ^∗^ *p* < 0.05 versus the blank group; ^^^ *p* < 0.05 versus the LPS group; ^#^versus the ISO group.

To elucidate the relationship between *β*2-AR and TLR4, after treatment with ISO or LPS, the protein level of *β*2-AR was significantly decreased; the TLR4 protein levels were significantly decreased with ISO treatment and remarkably increased with LPS challenge. Furthermore, TLR4 was decreased after stimulating with ISO and LPS compared with LPS (*p* < 0.05) (Figure 3(a), Fig.  S5). In the present report, we found that ISO stimulation attenuated the cell surface expression of *β*2-AR; this result was consistent with previous findings that ISO promoted *β*2-AR internalization from the cell membrane to endosomes. After LPS stimulation, ISO activated *β*2-AR function and decreased TLR4 expression to protect the endothelial barrier [5]. Additionally, the data show that ISO effectively decreased cell hyperpermeability and the overexpression levels of IL-6, IL-8, and TNF-*α* by LPS challenge; however, the effect of ISO was not obvious without LPS stimulation (Figures 3(b)–3(d)). In contrast, LPS promoted cell hyperpermeability and the expression of inflammatory factors. We were surprised to find that IL-17A expression was not significantly different among these groups (*p* > 0.05). These data demonstrate that ISO and LPS have opposite effects on *β*2-AR and TLR4 and that *β*2-AR and TLR4 have mutual negative regulation in LPS-induced receptor imbalance.

### 3.4. Effect of siRNA on *β*2-AR and TLR4 in HPMECs

**Figure 4:**
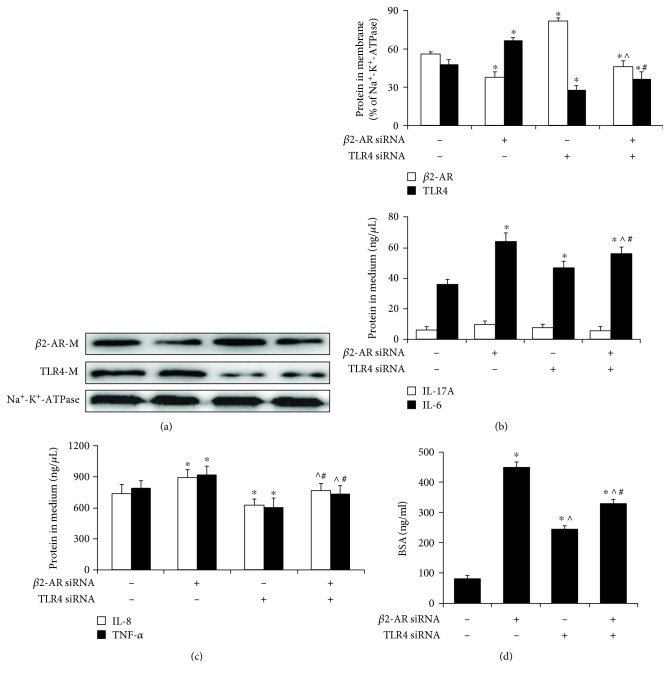
Knock-down of *β*2-AR and TLR4 in HPMECs. (a) The expression of membrane proteins after transfection with *β*2-AR siRNA and TLR siRNA. Quantification of protein levels by Western blotting. The representative blots show the expression of *β*2-AR (A, upper), TLR4 (A, middle), and Na^+^-K^+^-ATPase (A, lower). The histograms show the quantitative analysis of the levels of *β*2-AR and TLR4 normalized to Na^+^-K^+^-ATPase. (b, c) Expression of inflammatory factors (IL-6, IL-17A, IL-8, and TNF-*α*) after transfection with siRNA. (d) Effect of siRNA treatment on monolayer permeability. The data are the means ± SD (*n* = 3). ^∗^ *p* < 0.05 versus the NC; ^^^ *p* < 0.05, versus the *β*2-AR siRNA group, ^#^ *p* < 0.05, versus the TLR4 siRNA group.

Our previous data demonstrated that an agonist (ISO) enhanced *β*2-AR function to protect the endothelial barrier and inhibit the inflammatory reaction in LPS-induced injury. Based on these findings, we further explored the effect of siRNA on *β*2-AR and TLR4. The result revealed that the expression of cell surface *β*2-AR significantly increased, and TLR4 markedly decreased after silencing with TLR4 siRNA; in contrast, the expression of TLR4 significantly increased, and *β*2-AR markedly decreased after treatment with *β*2-AR siRNA, compared with the NC group (*p* < 0.05). Moreover, *β*2-AR and TLR4 expressions also were significantly decreased after treatment with *β*2-AR siRNA and TLR4 siRNA, compared with NC; however, *β*2-AR expression was slightly increased at cellular membrane silencing with *β*2-AR siRNA and TLR4 siRNA, compared to *β*2-AR siRNA-treated alone; similarly, TLR4 also was increased after silencing *β*2-AR and TLR4, compared to the TLR4 siRNA group (*p* < 0.05) (Figure 4(a), Fig.  S6). The results indicated that TLR4 siRNA attenuates the BSA diffusion coefficient in cells to protect the endothelial barrier and improves the inflammatory response (*p* < 0.05) (Figures 4(b)–4(d)). Furthermore, *β*2-AR siRNA caused the hyperpermeability of HPMECs and promoted the inflammatory response (*p* < 0.05), but IL-17A expression was not appreciably different among these groups (*p* > 0.05). Thus, these data confirmed that a receptor imbalance phenomenon exists between *β*2-AR and TLR4 in HPMECs.

## 4. Discussion

Rab proteins are key regulators of vesicle trafficking. Rab GTPase function is regulated by exchange cycles between the GDP-bound inactive forms and GTP-bound active forms [5, 6, 23]. Rab26 is a member of the Rab protein family, and similar to other Rab GTPases, it modulates anterograde receptor trafficking to the cell surface, receptor transport to lysosomes for degradation, and cell membrane receptor internalization into endosomes following agonist stimulation [4, 19]. Previous studies have shown that Rab26 inactivation induces the mistrafficking of GPCRs, which leads to receptor dysfunction that is directly linked to cell function disorders; other studies have shown that treatment with Rab26 siRNA destroys barrier function and that Rab26 even promotes adherens junction integrity in an autophagy/macroautophagy-dependent manner in ALI/ARDS [9, 16, 22]. This report showed that the Rab26 expression is reduced by treatment with the inflammatory mediator LPS, which causes cell inflammation and cell hyperpermeability. Furthermore, the suppression of Rab26 function aggravates the inflammatory reaction and increases cell permeability after LPS induction. In contrast, promoting Rab26 function prevents LPS-induced endothelial barrier dysfunction and inflammation. Therefore, Rab26 is a crucial target for reducing LPS-induced damage in ALI. These results are in line with those of previous studies.

*β*2-AR is one type of GPCRs that plays key roles in protecting the endothelial barrier. Rab1 coordinates *β*2-AR transport from the endoplasmic reticulum (ER) to the Golgi body in permeability regulation in PMECs [4]; Rab5 GTPase regulates *β*-AR internalization to protect the endothelial barrier [5]. However, the role of Rab26 in transporting *β*-AR remains poorly understood. Rab26 mediates the transport of *α*2-AR from the Golgi to the plasma membrane in H293 cells, thereby regulating a series of biological cellular processes [9]. In this report, we first demonstrated that Rab26 significantly increased the expression of *β*2-AR on the plasma membrane, which provided preliminary evidence that Rab26 transports intracellular *β*2-AR to the cell membrane. In addition, the TLR4 signaling pathway, which mainly responds to LPS-induced inflammation, plays a crucial role in ALI [24]. Similar to the results of these latter studies, our results indicated that Rab26 is required for the cell surface expression of TLR4 to further regulate the inflammatory reaction and cell permeability. Interestingly, we discovered that Rab26 has opposite effects on these two receptors; Rab26 has a positive regulatory effect on *β*-AR but negatively regulated TLR4. Thus, we strongly suspected a phenomenon of receptor imbalance because of the crosstalk between these two receptors.

Increasing the crosstalk between *β*2-AR and TLR4 via agonists (ISO or LPS) and siRNA-mediated depletion promoted the permeability of HPMECs and regulated the production of inflammatory cytokines. Indeed, the *β*-AR agonist salbutamol may stimulate epithelial repair in ARDS [25], specifically by upregulating matrix metalloproteinase-9 in ARDS patients [26] and reducing the incidence of pneumonia and some alveolar inflammation [27]. In the present study, we observed that ISO activated *β*2-AR function and decreased TLR4 expression to protect the endothelial barrier and alleviate LPS-induced inflammation. Furthermore, TLR4 activation by LPS induces cell hyperpermeability and inflammation and weakens the protective effect of *β*2-AR on the endothelial barrier. These data were consistent with those in previous studies showing that the LPS-induced TLR4 signaling pathway triggers a systemic inflammatory response during endothelial cell apoptosis and increases cultured endothelial cell permeability and that LPS administration in various models induces profound vascular leakage in vivo [28–30].

Herein, we found that *β*2-AR activation or silencing TLR4 improved LPS-induced hyperpermeability in HPMECs. Another study also has shown that DTA0118, an inhibitor of TLR4, prevents the LPS-induced lung epithelial cell injury by inhibiting the secretion of proinflammatory cytokines, downregulating TLR4, upregulating I*κ*B*α*, and preventing apoptosis [31]. Consistent with present research, we found that TLR4 siRNA played a protective role in attenuating the permeability and inflammatory response, which suppressed TLR4 downstream MyD88-NF-*κ*B pathway [5, 16]. On the contrary, our data demonstrates that *β*2-AR siRNA increased the permeability and inflammation of HPMECs. This effect may be involving inhibition of *β*2-AR downstream MAPK, ERK_1/2_, and cAMP signal transduction pathways. Furthermore, 2-choloroethyl ethyl sulfide, an inhibitor of *β*2-AR, reduces the responsiveness of *β*2-AR, induces generation of ROS, upregulates TNF-*α*, enhances infiltration of inflammatory cells, and increases stress-related endogenous catecholamine in lung injury [32]. Interestingly, we found that silencing *β*2-AR and TLR4 attenuated hyperpermeability and inflammatory mediator production compared to *β*2-AR knocking down alone in HPMECs. These data suggests that *β*2-AR activation or TLR4 inhibition is the strategy for protecting endothelial barrier from LPS-induced endothelium injury.

This study found that the imbalance between *β*2-AR and TLR4 is involved in LPS-induced endothelial barrier dysfunction. Basal Rab26 maintains the balance between the two receptors through modulating cell surface receptor expression, which may be a potential therapeutic target for preventing endothelial barrier disruption and vascular endothelial inflammation. Nevertheless, in the present research, we observed a phenomenon of receptor imbalance related to Rab26 on cell levels, but the mechanism how Rab26 regulates the balance between *β*2-AR and TLR4 and studies in an animal model need further investigation.

In conclusion, our studies provided evidence that Rab26 is involved in the regulation of LPS-induced endothelial barrier dysfunction. This function of Rab26 is likely mediated through maintaining the balance between the *β*2-AR and TLR4. These studies suggest that Rab26 may be a potential therapeutic target for diseases involving endothelial barrier dysfunction.

## Data Availability

The data used to support the findings of this study are included within the article. The FACS analysis of the time- and dose-dependent cell transfection efficiency data used to support the findings of this study is included within the supplementary information.

## Supplementary Materials

Supplementary Materials Fig.S1: the maps of plasmid vectors: (A) pDsRed-monomer-C1 vector and (B) HA-pcDNA3.1- vector. Fig.S2: FACS analysis of the transfection efficiency of DsRed-tagged Rab26 plasmid. (A) Time- and dose-dependent cell transfection efficiencies of the DsRed-tagged Rab26^WT^ plasmid in HPMECs. HPMECs were incubated with the transfection reagent (2 *μ*L, 4 *μ*L, 6 *μ*L, and 8 *μ*L) and DsRed-tagged Rab26^WT^ plasmid (1 *μ*g) at various periods of time (0 h, 24 h, 48 h, or 72 h). (B) Transfection efficiency of the DsRed-tagged Rab26 plasmid by FACS. HPMECs were transfected with the ViaFect™ transfection reagent (4 *μ*L) and plasmid (1 *μ*g) for 72 h. Blank, DsRed vector (mock), DsRed-tagged Rab26 wild type (Rab26^WT^), DsRed-tagged Rab26 Q123L (Rab26^QL^), and DsRed-tagged Rab26 N177I (Rab26^NI^). The data are the means ± SD (*n* = 3). ^^^ *p* < 0.05 versus the groups incubated for 24 h; ^∗^ *p* < 0.05 versus the 48 h group. Fig.S3: original full-size Western blots used for Figure 1(b). (A) Figure 1(b). (B-D) The WB full membranes of Rab26. The molecular mass (MW) is 28 kDa. (E-G) The WB full membranes of GAPDH. The molecular mass (MW) is 37 kDa. The data are the means ± SD (*n* = 3). ^∗^ *p* < 0.05 versus the control group; ^^^ *p* < 0.05 versus the mock group. Fig.S4: original full-size Western blots used for Figure 2(a). (A) Figure 2(a). (B-D) The full WB membranes of *β*2-AR. The molecular mass (MW) is 45 kDa. (E-G) The full WB membranes of TLR4. The molecular mass (MW) is 97 kDa. (H-J) The full WB membranes of Na^+^-K^+^-ATPase. The molecular mass (MW) is 117 kDa. The data are the means ± SD (*n* = 3). ^∗^ *p* < 0.05 versus the control group; ^^^ *p* < 0.05 versus the mock group. Fig.S5: original full-size Western blots used for Figure 3(a). (A) Figure 3(a). (B-D) The full WB membranes of *β*2-AR. The molecular mass (MW) is 45 kDa. (E-G) The full WB membranes of TLR4. The molecular mass (MW) is 97 kDa. (H-J) The full WB membranes of Na^+^-K^+^-ATPase. The molecular mass (MW) is 117 kDa. The data are the means ± SD (*n* = 3). ^∗^ *p* < 0.05 versus the blank group; ^^^ *p* < 0.05 versus the LPS group; ^#^versus the ISO group. Fig.S6: original full-size Western blots used for Figure 4(a). (A) Figure 4(a). (B-D) The full WB membranes of *β*2-AR. The molecular mass (MW) is 45 kDa. (E-G) The full WB membranes of TLR4. The molecular mass (MW) is 97 kDa. (H-J) The full WB membranes of Na^+^-K^+^-ATPase. The molecular mass (MW) is 117 kDa. The data are the means ± SD (*n* = 3). ^∗^ *p* < 0.05 versus the NC; ^^^ *p* < 0.05, versus the *β*2-AR siRNA group, ^#^ *p* < 0.05, versus the TLR4 siRNA group.
