# Supplementary material for: Endothelial Cell Inflammation and Barriers Are Regulated by the Rab26-Mediated Balance between β2-AR and TLR4 in Pulmonary Microvessel Endothelial Cells
Source: Mediators Inflamm. 2019 Apr 28;2019:7538071. doi: 10.1155/2019/7538071 (PMC6512073; doi:10.1155/2019/7538071)
Supplement: Supplementary Materials — Fig.S1: the maps of plasmid vectors: (A) pDsRed-monomer-C1 vector and (B) HA-pcDNA3.1- vector. Fig.S2: FACS analysis of the transfection efficiency of DsRed-tagged Rab26 plasmid. (A) Time- and dose-dependent cell transfection efficiencies of the DsRed-tagged Rab26WT plasmid in HPMECs. HPMECs were incubated with the transfection reagent (2 μL, 4 μL, 6 μL, and 8 μL) and DsRed-tagged Rab26WT plasmid (1 μg) at various periods of time (0 h, 24 h, 48 h, or 72 h). (B) Transfection efficiency of the DsRed-tagged Rab26 plasmid by FACS. HPMECs were transfected with the ViaFect™ transfection reagent (4 μL) and plasmid (1 μg) for 72 h. Blank, DsRed vector (mock), DsRed-tagged Rab26 wild type (Rab26WT), DsRed-tagged Rab26 Q123L (Rab26QL), and DsRed-tagged Rab26 N177I (Rab26NI). The data are the means ± SD (n = 3). ^ p < 0.05 versus the groups incubated for 24 h; ∗ p < 0.05 versus the 48 h group. Fig.S3: original full-size Western blots used for Figure 1(b). (A) Figure 1(b). (B-D) The WB full membranes of Rab26. The molecular mass (MW) is 28 kDa. (E-G) The WB full membranes of GAPDH. The molecular mass (MW) is 37 kDa. The data are the means ± SD (n = 3). ∗ p < 0.05 versus the control group; ^ p < 0.05 versus the mock group. Fig.S4: original full-size Western blots used for Figure 2(a). (A) Figure 2(a). (B-D) The full WB membranes of β2-AR. The molecular mass (MW) is 45 kDa. (E-G) The full WB membranes of TLR4. The molecular mass (MW) is 97 kDa. (H-J) The full WB membranes of Na+-K+-ATPase. The molecular mass (MW) is 117 kDa. The data are the means ± SD (n = 3). ∗ p < 0.05 versus the control group; ^ p < 0.05 versus the mock group. Fig.S5: original full-size Western blots used for Figure 3(a). (A) Figure 3(a). (B-D) The full WB membranes of β2-AR. The molecular mass (MW) is 45 kDa. (E-G) The full WB membranes of TLR4. The molecular mass (MW) is 97 kDa. (H-J) The full WB membranes of Na+-K+-ATPase. The molecular mass (MW) is 117 kDa. The data are the means ± SD (n = 3). ∗ p < 0.05 [file 7538071.f1.pdf]

Endothelial cell inflammation and barriers are regulated by the Rab26-mediated balance between β_2-AR and TLR4 in pulmonary microvessel endothelial cells

Huaping Chen[1,#], Ming Yuan[1,#], Chunji Huang[2], Zhi Xu[1], Mingchun Li[1], Chun Zhang[1], Zhan Gao[1], Mingzhou Zhang[1], Jiancheng Xu[1], Hang Qian[1], Jiegen You[3,*], Binfeng He[1], Guansong Wang[1], Mingdong Hu[1, *]

[1] Institute of Respiratory Diseases, Xinqiao Hospital, Third Military Medical University, Chongqing 400037, China

[2] Basic Medical College, Third Military Medical University, Chongqing 400038, China

[3] Jiangxi Academy of Medical Sciences, Nanchang City, Jiangxi Province 330006, China

[#] Contributed equally to this work

Correspondence should be addressed: * Mingdong Hu, huhanshandd@aliyun.com; and *Jiegen You, yjg700330@163.com

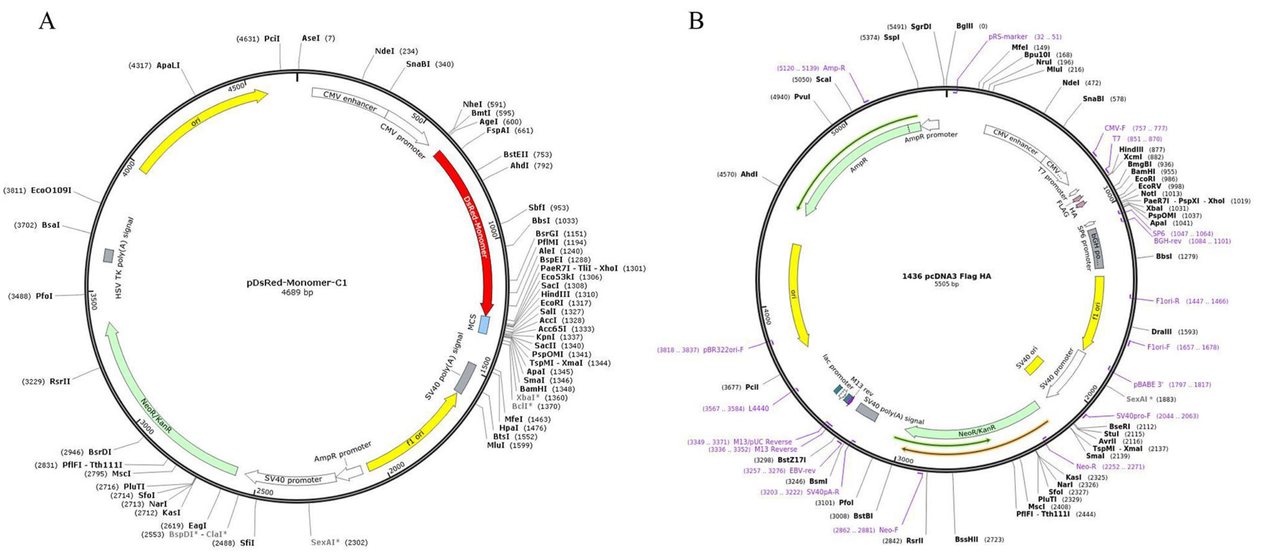

Fig.S1. The maps of plasmid vectors. (A) pDsRed-monomer-C1 vector. (B) HA-pcDNA3.1- vector.

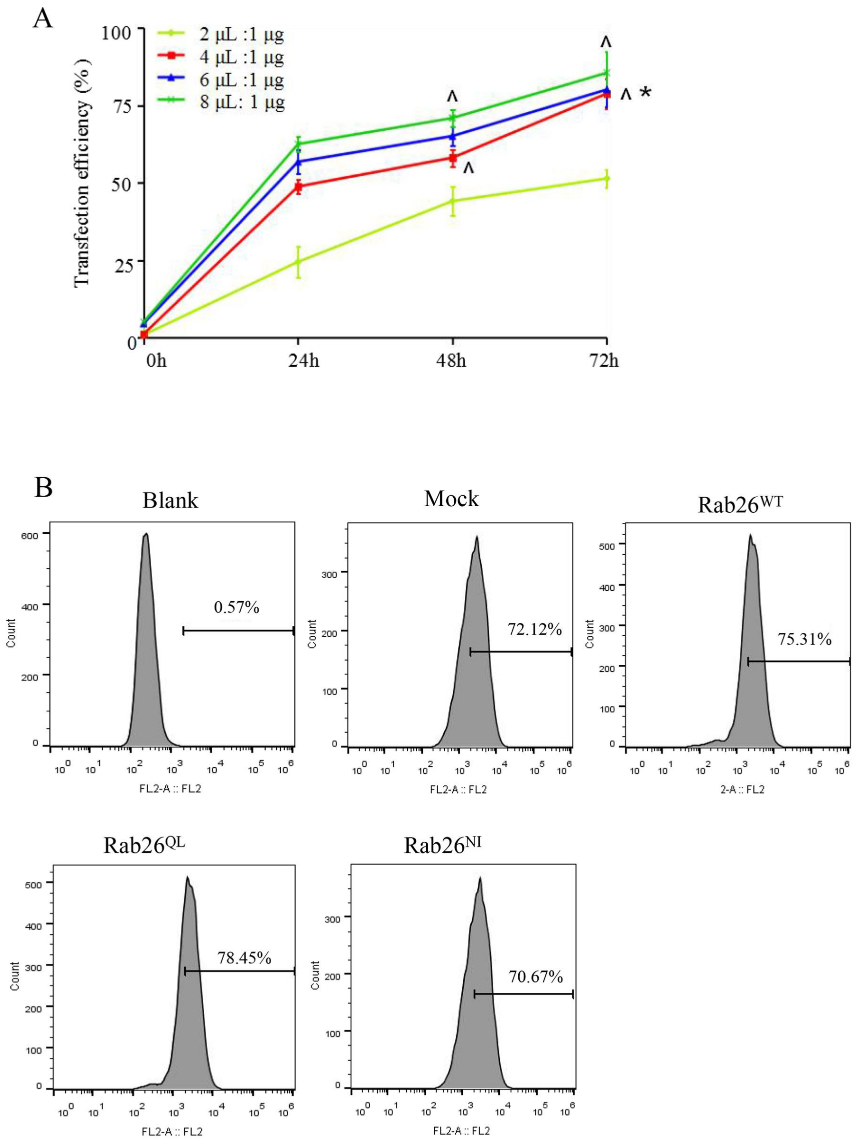

Fig.S2. FACS analysis the transfection efficiency of DsRed-tagged Rab26 plasmid. (A). Time- and- dose dependent cell transfection efficiency of the DsRed-tagged Rab26^{WT} plasmid in HPMECs. HPMECs were incubated with transfection reagent (2μL, 4 μL, 6μL, 8μL) and DsRed-tagged Rab26^{WT} plasmid (1 μg) at various periods of time (0 h, 24 h, 48 h, or 72 h). (B). Transfection efficiency of the DsRed-tagged Rab26 plasmid by FACS. HPMECs were transfected with ViaFectTM transfection reagent (4 μL) and plasmid (1 μg) for 72 h. Blank, DsRed vector (Mock), DsRed-tagged Rab26 wild-type (Rab26^{WT}), DsRed-tagged Rab26Q123L (Rab26^{QL}), DsRed-tagged Rab26N177I (Rab26^{NI}). The data are the means ± SD (n = 3). ^, $p < 0.05$ versus the groups incubated for 24 h; *, $p < 0.05$ versus the 48 h group.

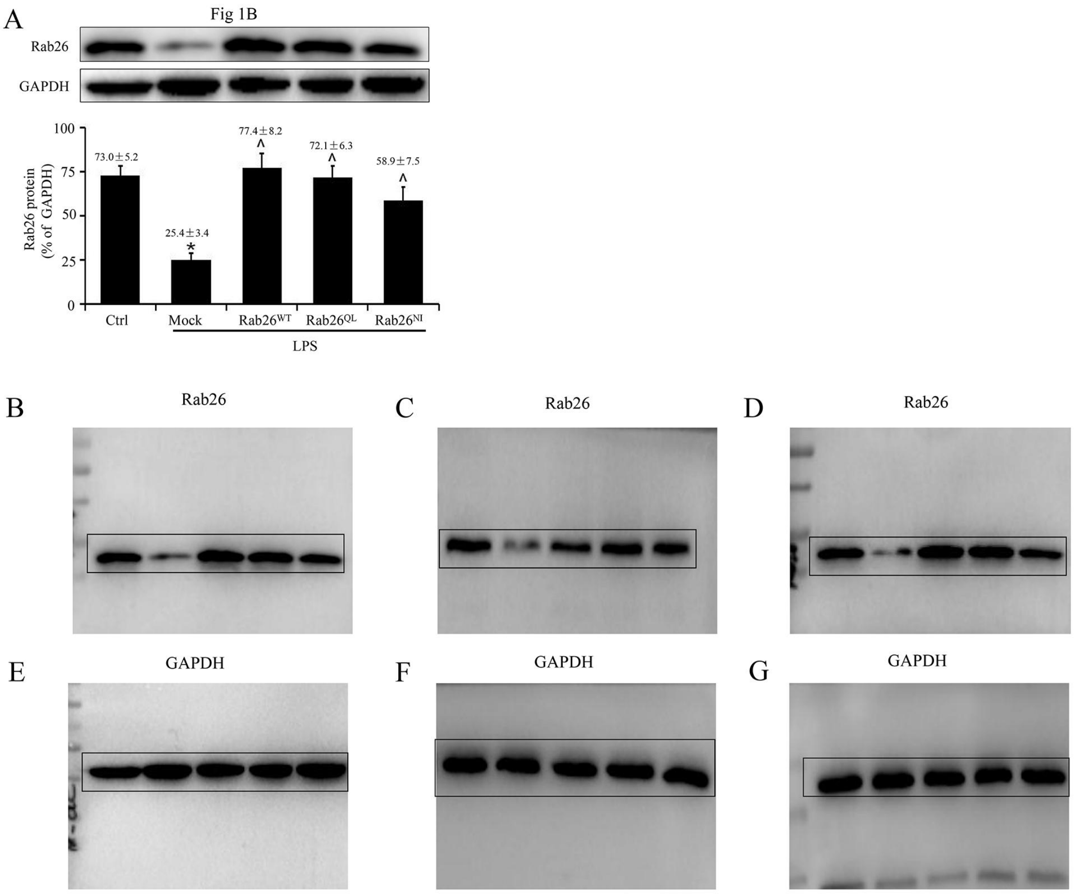

Fig.S3. Original full-size western blots used for Figure 1B. (A) Fig. 1B. (B-D) The WB full membranes of Rab26. The molecular mass (MW) is 28 kDa. (E-G) The WB full membranes of GAPDH. The molecular mass (MW) is 37 kDa. The data are the means ± SD (n = 3). *, $p < 0.05$ versus the control group; ^, $p < 0.05$ versus the mock group.

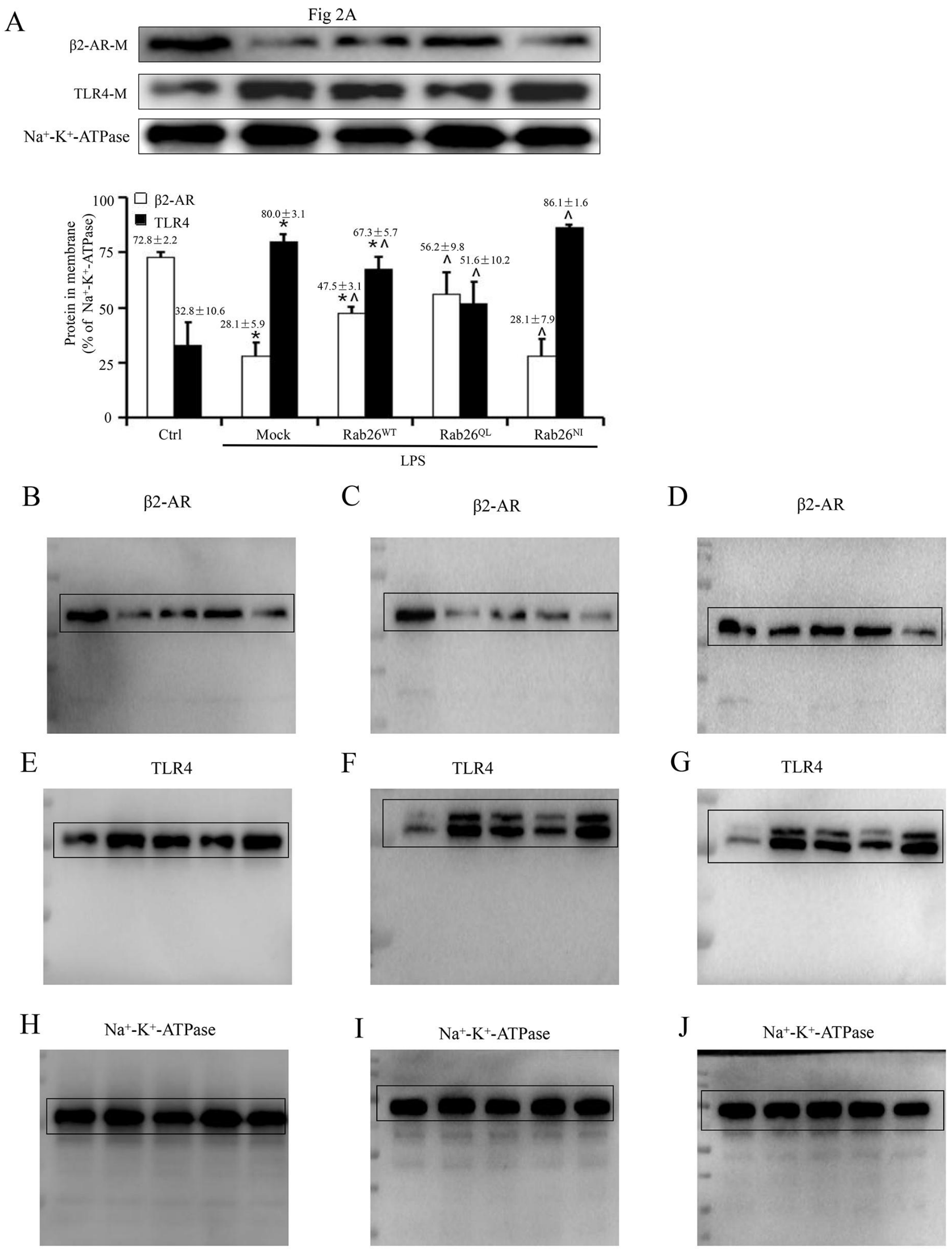

Fig.S4. Original full-size western blots used for Figure 2A. (A) Fig. 2A. (B-D) The full WB membranes of β_2-AR. The molecular mass (MW) is 45 kDa. (E-G) The full WB membranes of TLR4. The molecular mass (MW) is 97 kDa. (H-J) The full WB membranes of Na^+-K^+-ATPase. The molecular mass (MW) is 117 kDa. The data are the means ± SD (n = 3). *, $p < 0.05$ versus the control group; $^\wedge$, $p < 0.05$ versus the mock group.

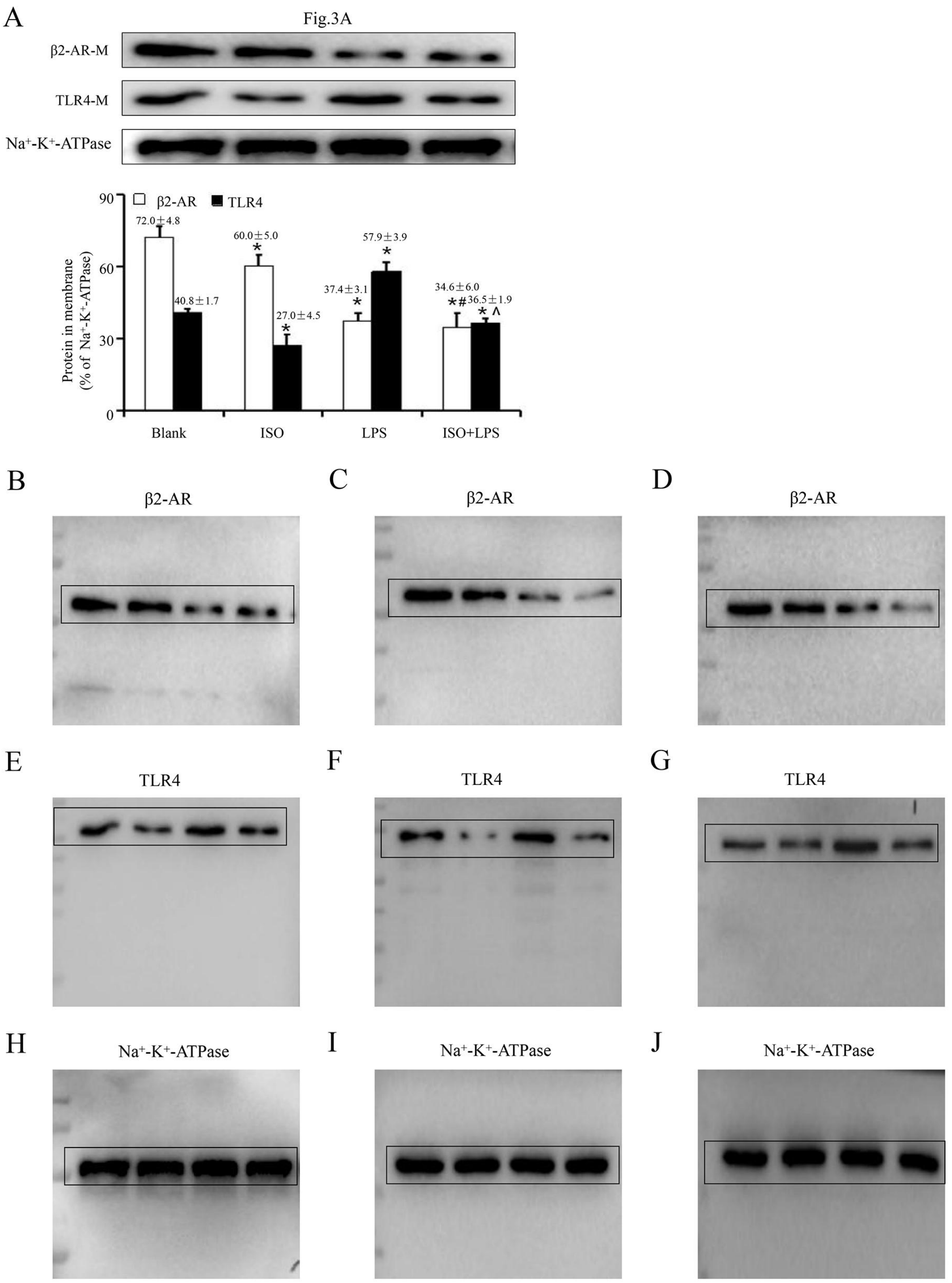

Fig.S5. Original full-size western blots used for Figure 3A. (A) Fig. 3A. (B-D) The full WB membranes of β_2-AR. The molecular mass (MW) is 45 kDa. (E-G) The full WB membranes of TLR4. The molecular mass (MW) is 97 kDa. (H-J) The full WB membranes of Na^+-K^+-ATPase. The molecular mass (MW) is 117 kDa. The data are the means ± SD (n = 3). *, $p < 0.05$ versus the blank group; $^\wedge$, $p < 0.05$ versus the LPS group; $^\#$, versus the ISO group.

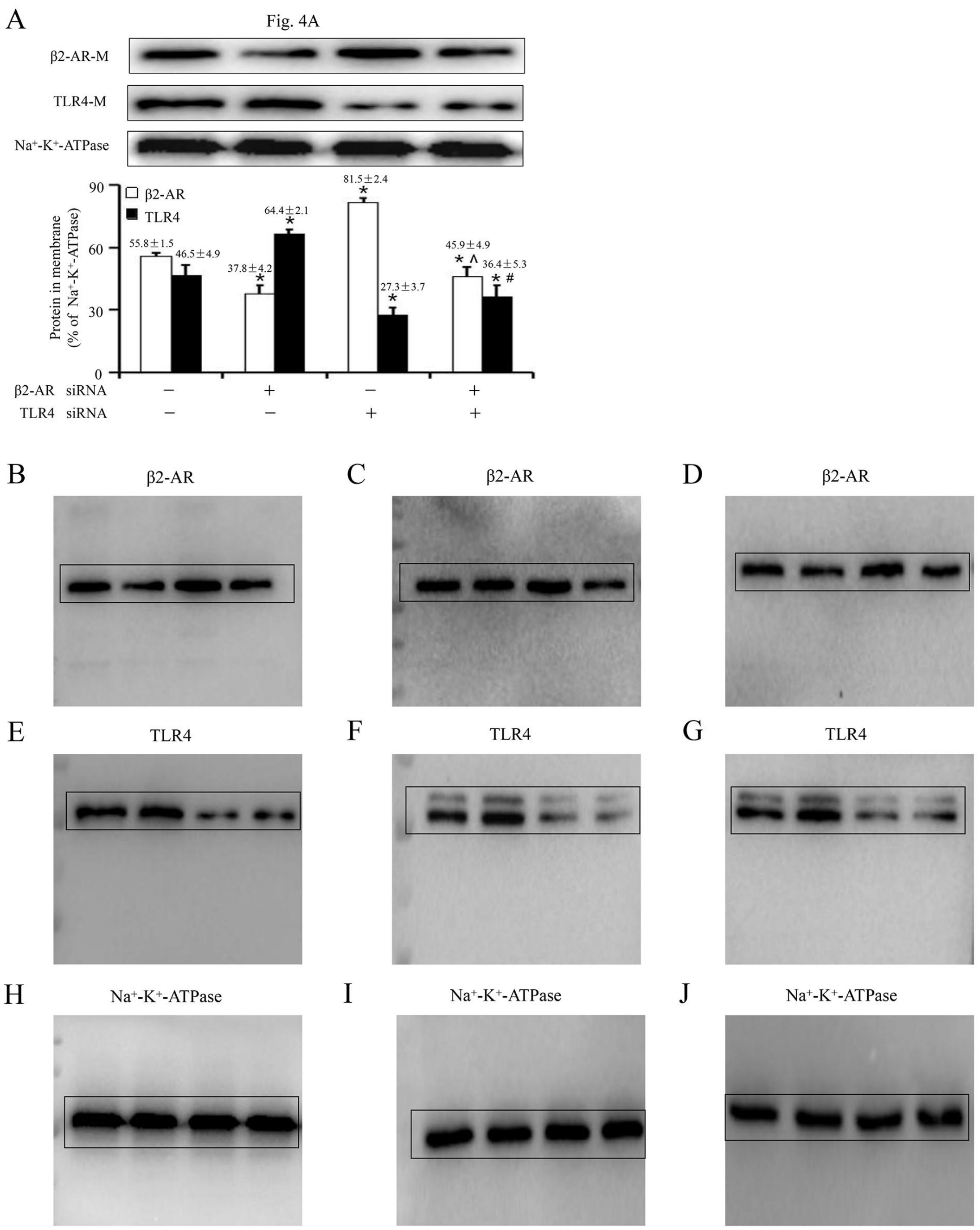

Fig.S6. Original full-size western blots used for Figure 4A. (A) Fig. 4A. (B-D) The full WB membranes of β2-AR. The molecular mass (MW) is 45 kDa. (E-G) The full WB membranes of TLR4. The molecular mass (MW) is 97 kDa. (H-J) The full WB membranes of Na^+-K^+-ATPase. The molecular mass (MW) is 117 kDa. The data are the means ± SD (n = 3). *, $p < 0.05$ versus the NC; $^\wedge$, $p < 0.05$, versus the β2-AR siRNA group, $^\#$, $p < 0.05$, versus the TLR4 siRNA group.
